# A Retrospective Analysis of Oral Langerhans Cell Histiocytosis in an Iranian Population: a 20-year Evaluation

**Published:** 2015-09

**Authors:** Saede Atarbashi Moghadam, Ali Lotfi, Batool Piroozhashemi, Sepideh Mokhtari

**Affiliations:** a Dept. of Oral and Maxillofacial Pathology, School of Dentistry, Shahid Beheshti University of Medical Sciences, Tehran, Iran.; b Dentist, Private Practice, Tehran, Iran.

**Keywords:** Langerhans Cell Histiocytosis, Eosinophilic Granuloma, Oral, Prevalence

## Abstract

**Statement of the Problem:**

Langerhans cell histiocytosis is a rare disease with unknown pathogenesis and is characterized by local or disseminated proliferation of Langerhans cells. There is no previous investigation on prevalence of oral Langerhans cell histiocytosis in Iranian population.

**Purpose:**

The purpose of this study was to assess the relative frequency of oral Langerhans cell histiocytosis in an Iranian population and to compare the data with previous reports.

**Materials and Method:**

Pathology files of Oral and Maxillofacial Pathology Department of Dental School of Shahid Beheshti University of Medical Sciences from 1992 to 2012 were searched for cases recorded as oral Langerhans cell histiocytosis. A total number of 20 cases were found and the clinical information of patients was recorded.

**Results:**

The relative frequency of oral Langerhans cell histiocytosis was 0.34% and the most common location was the posterior mandible. In addition, the mean age of patients was 27 years and there was a definite male predominance. Most lesions were localized and tooth mobility was the most common oral presentation.

**Conclusion:**

In Iranian population as in many other countries, the relative frequency of oral Langerhans cell histiocytosis is low. Moreover, tooth mobility and periodontal lesions are the frequent early signs of disease. Therefore, in patients with periodontal problems, good oral health, and no response to the treatment; Langerhans cell histiocytosis must be considered. Additionally, although most cases of oral Langerhans cell histiocytosis are localized, systemic involvement must also be considered and dental professionals have an important role in early detection of the disease.

## Introduction

Langerhans cell histiocytosis (LCH) is caused by an uncontrolled pathogenic clonal proliferation of dendritic cells with Langerhans cell characteristics.(1) No definite etiology has been identified for this disease and it can be triggered by environmental agents and viruses, in particular Epstein-Barr virus.(2)

Three clinical subtypes of LCH are recognized. 

The unifocal subtype (single system, single site), previously referred to as eosinophilic granuloma, usually affects the bones, lymph nodes, or lungs as the primary sites.(3) The multifocal subtype (single system, multiple sites) affects several sites in any particular organ system. Formerly this subtype was recognized as Hand- Schuller- Christian disease with bone lesions, diabetes insipidus, and exophthalmos.(4) The multiple-organ-system subtype, previously referred to as disseminated histiocytosis or Letterer–Siwe disease, affects multiple sites in different organ systems and is seen in the first year of life and has the worst prognosis.(4)

The jaws are affected in 10% to 20% of all cases. Dull pain and tenderness often accompany bone lesions. Bone destruction and tooth loosening may clinically resemble severe periodontitis. The lesions in periapical sites may mimic periapical inflammatory lesions. The involved gingival tissues are often inflamed, hyperplastic, or ulcerated. Oral mucosal lesions in form of submucosal nodules, ulcers, and leukoplakia have also been described.(5-6)

Since there was no previous research about oral LCH in Iranian population, we assessed the relative frequency of oral LCH and compared the results with other countries. 

## Materials and Method

The files of oral and maxillofacial pathology department in Dental School of Shahid Beheshti University of Medical Sciences, Tehran, Iran, served as the source of materials during a 20-year-period from 1992 to 2012. All lesions diagnosed as oral LCH were subjected to microscopic re-evaluation. Then, immunohistochemical examination with CD1a protein was performed to confirm the diagnosis. Information including patients’ age and gender, lesions’ location, and patients’ clinical symptoms was also recorded. The Chi-square test was used and the results with *p*< 0.05 were considered significant.

## Result

During this period, 5744 specimens were referred to the oral pathology department. Twenty cases (0.34% of all cases) were identified as LCH. The mean age of patients was 27 years (ranged from 3 months to 51 years) and the majority of patients aged between 21 and 30 years old. 15 lesions had occurred in males and 5 in females (ratio 3:1). Chi-square test showed a significant difference between males and females. In addition, all the lesions were intraosseous. 45% of all lesions had occurred in the mandible, 45% in both mandible and maxilla, and only 5% in the maxilla (Table 1). The ratio of mandibular to maxillary involvement was statistically significant (9 to 1). The posterior regions (especially in mandible) were the predominant sites of occurrence.

**Table 1:** Location of oral involvement in LCH cases

**Location of Jaw Lesions**	**Number of Cases**
Posterior mandible	6 (30%)
Diffuse lesions in maxilla and mandible	4 (20%)
Posterior maxilla and mandible	4 (20%)
Posterior and anterior mandible	3 (15%)
Posterior maxilla	1 (5%)
Unknown	2 (10%)
Total number of jaw lesions	20 (100%)

The most common oral presenting symptom was tooth mobility (50%), followed by pain (35%) and swelling (35%) (Table 2).

**Table 2:** Various presentation forms of LCH in oral cavity lesions

**Clinical presentation**	**Number of cases**
Tooth mobility	10 (50%)
Pain and tenderness	7 (35%)
Expansion	7 (35%)
Ulceration	4 (20%)
Granulomatous lesion	2 (10%)
Hyperkeratotic lesion	1 (5%)

Fifteen patients (75%) had lesions limited to the oral cavity and were diagnosed as eosinophilic granuloma. The remaining 5 patients (25%) had the disseminated form of disease (Table 3).

**Table 3:** Frequency of localized and diffuse lesions in LCH cases

**Being Diffuse or Localized**	**Number of Cases**
Localized lesions	14
Diffuse lesions	2 : Hand–Schuller–Christian
	1 : Letterer–Siwe
	2 : Not Classified
Unknown	1
Total number of cases	20

**Figure 1:**
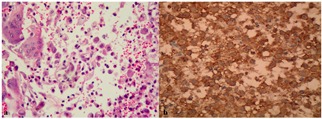
a: Giant cells may be present in microscopic features of LCH (H&E, 400X) b: Immunohistochemical examination shows strong membranous expression of CD1a in Langerhans cells (IHC, × 400)

Two cases had the triad of bone lesions, diabetes insipidus and exophthalmos and were diagnosed as Hand- Schuller–Christian disease. One case was a child with diffuse involvement and was diagnosed as Letterer- Siwe disease. There was no relationship between gender and the pattern of the disease (localized or disseminated) (*p*=0.13), gender and location (*p*=0.2), gender and age (*p*= 0.85) and age and the pattern of the disease (*p*= 0.22). Microscopically, all cases demonstrated sheets of histiocyte-like cells with varying amounts of eosinophils, lymphocytes and giant cells (Figure 1a). Furthermore, immunohistochemical examination showed strong positivity (membranous brown staining) for CD1a protein in all cases (Figure 1b).

## Discussion

Epidemiologic studies provide important details on the disease occurrence and its microscopic features in various countries.(7-8) These data will improve detection of high risk populations and help determine various responsible factors. The records available at oral and maxillofacial pathology centers are major information sources for epidemiologic studies of oral lesions.(9)

Although there is no previous research about oral LCH in Iranian population, our findings indicated that LCH prevalence in Iran was similar to other countries. In this research, oral LCH accounted for 0.34% of all oral pathology cases, which was similar to other investigations.(10-12) Previous epidemiologic studies have reported 17 cases of oral LCH during a period of 40 years, 4 cases in a period of 16 years and 3 cases in a 30-year period,(10-12) indicating the low prevalence of the disease.

LCH can affect any age group; however, it is more frequent in children.(10) Nevertheless, the mean age of patients in our research was 27 years. In fact, bony lesions of LCH usually occur in patients above 20. Therefore, the mean age of patients with jaw involvement is higher than other groups.

In this study, oral LCH was more frequent in males, generally affecting the posterior region of mandible. This was in agreement with other investigations.(4, 13)

The presenting signs and symptoms of oral LCH may be pain, swelling, oral mucosal ulceration, periodontal problems, non-healing extraction sites and granulomatous or hyperkeratotic lesions.(10, 14-15) These various presentations of oral LCH is due to the fact that either jaws or oral mucosa are involved. Oral LCH in this case series most frequently presented with tooth mobility, pain, and swelling.

Oral LCH lesions are localized or the earliest sign of a disseminated disease.(3) Various frequencies of disseminated form (10-66%) have been reported in the articles.(15-16) Most patients in our research (75%) had localized lesions and the remaining minority (25%) had systemic involvement. Since oral lesions may be the initial manifestations of a systemic involvement, dental professionals have an important role in early detection of the disease.(17) Once the diagnosis of LCH is established, the patient should be referred by the dentist to an internist for a thorough physical examination, particularly in lymph nodes and abdominal organs to rule out a more disseminated disease. It must be noted that any delay in diagnosis of LCH may lead to a poor response to the treatment.

## Conclusion

Like many other countries, the relative frequency of oral Langerhans cell histiocytosis is low in Iranian population and the great majority of cases occur in males. In addition, tooth mobility and periodontal lesions are frequently the early signs of disease. Therefore, in patients with periodontal problems, good oral health, and no response to the treatment, LCH must be considered. Although most cases of oral LCH are localized, a thorough examination should be performed to rule out any systemic involvement. 
